# The Power of Thermosonication on Quality Preservation and Listeria Control of Blueberry Juice

**DOI:** 10.3390/foods13223564

**Published:** 2024-11-07

**Authors:** Eleonora Panaro, Teresa R. S. Brandão, Cristina L. M. Silva, Fátima A. Miller

**Affiliations:** CBQF—Centro de Biotecnologia e Química Fina—Laboratório Associado, Escola Superior de Biotecnologia, Universidade Católica Portuguesa, Rua Diogo Botelho 1327, 4169-005 Porto, Portugal; eleonora.panaro96@gmail.com (E.P.); tbrandao@ucp.pt (T.R.S.B.); clsilva@ucp.pt (C.L.M.S.)

**Keywords:** fruit juices, ultrasounds, phenolics, anthocyanins, inactivation kinetics, thermal treatment

## Abstract

Due to the increasing consumer demand for healthy, beneficial foods, natural fruit juices have gained popularity for their rich nutritional value and appealing flavor. However, traditional thermal processing can compromise these quality attributes. This study investigates using pulsed thermosonication, a novel mild thermal processing method, on *Listeria innocua* inactivation in blueberry juice, chosen for its high phenolic and anthocyanin content. Ultrasonication was applied at 60% and 100% amplitudes combined with heat treatments at 45 °C and 55 °C and compared to control heat treatments. The Weibull model effectively described the inactivation kinetics, showing that the thermosonicated samples required significantly shorter times (1 and 25 min) for a 5-log reduction compared to the heated samples (10 and 60 min). While pH, total soluble solids, and water activity remained unaffected, color parameters improved, and the best retention of phenolics and anthocyanins was observed at 100% amplitude and 45 °C. Rheological properties were unchanged. The findings demonstrate that thermosonication at milder temperatures is more effective than conventional heat treatment for microbial inactivation and quality retention in blueberry juice, suggesting it is a superior processing method for preserving fruit juices’ nutritional and sensory attributes.

## 1. Introduction

In recent years, consumer preferences have shifted notably toward healthier and more natural food products. This trend has driven an increased demand for fruit juices, recognized as essential sources of vitamins, minerals, and bioactive compounds necessary for maintaining good health. Blueberry juice has gathered substantial attention due to its rich antioxidant content and related health benefits, including anti-inflammatory, anticancer, and antidiabetic properties [1,2]. However, traditional thermal pasteurization methods used to ensure the microbiological safety of fruit juices often lead to the loss of heat-sensitive nutrients and a reduction in sensory quality.

To address these challenges, innovative non-thermal processing technologies are being explored. One such technology is thermosonication, which combines the mechanical effects of ultrasound waves with mild heat treatment. This method has shown promise in enhancing food safety by achieving significant microbial inactivation while preserving the nutritional and sensory attributes of the juice [3]. Ultrasound treatment, particularly when combined with heat, disrupts microbial cells through cavitation and thermal effects, leading to efficient pathogen reduction without the extensive nutrient loss associated with conventional pasteurization.

Blueberries, belonging to the *Vaccinium* genus, are rich in anthocyanins, flavonoids, and phenolic acids, contributing to their vibrant color and potent antioxidant capacity [4]. The high antioxidant content makes blueberry juice desirable for health-conscious consumers [5]. However, the preservation of these bioactive compounds during processing is critical. Traditional thermal treatments can negatively impact these sensitive compounds, reducing the overall health benefits of the juice.

Previous studies on the application of ultrasound in fruit juice processing have demonstrated its potential to improve microbial safety and extend shelf life while maintaining quality. High-power ultrasound alone, however, often requires supplementary treatments, as a single ultrasound application can be insufficient for achieving significant microbial inactivation [6]. Mild heat treatments or hurdle technology combining ultrasonication with additional non-thermal methods are promising due to their scalability, energy efficiency, and minimal impact on juice quality [7]. For instance, research on various fruit juices, including orange [8,9,10], apple [11], and cantaloupe melon [12], has indicated that thermosonication can achieve effective microbial inactivation with minimal nutrient loss. However, the specific effects of thermosonication on blueberry juice remain underexplored, particularly concerning the inactivation of pathogenic microorganisms such as *Listeria monocytogenes* or its surrogate *L. innocua*.

*L. monocytogenes* is a foodborne pathogen that seriously threatens public health, particularly in low-acid fruit juices where it can survive and proliferate. However, due to safety concerns associated with handling *L. monocytogenes* in a laboratory environment, *L. innocua* was selected as a non-pathogenic surrogate in this study. *L. innocua* is widely recognized for its similar physiological characteristics to *L. monocytogenes*, particularly in response to environmental stresses such as heat and sonication. This makes it an ideal model for evaluating microbial inactivation under food processing conditions, ensuring that the results are both safe to obtain and relevant for assessing the potential reduction of *Listeria* in blueberry juice. Ensuring the inactivation of *Listeria* in blueberry juice is crucial for consumer safety. While high-power ultrasound alone has shown limited effectiveness in achieving the desired 5-log microbial reduction, combining ultrasound with mild heat (thermosonication) holds promise for enhanced microbial inactivation. A recent similar study on kiwi juice demonstrated the high effectiveness of thermosonication in achieving a significant 5-log-cycle reduction in *L. innocua* while successfully retaining the juice’s quality [3].

This study aims to evaluate the impact of pulsed thermosonication on the survival of *Listeria* in blueberry juice. Additionally, the research will assess the effects of this treatment on the physicochemical attributes, rheological properties, and retention of bioactive compounds in the juice. By comparing different amplitudes and temperatures of pulsed thermosonication with conventional heat treatments, this study seeks to identify optimal processing conditions that ensure safety and quality preservation in blueberry juice.

## 2. Materials and Methods

### 2.1. Juice Preparation

Packets of deep-frozen whole blueberries (*Vaccinium myrtillus* L.) were purchased in Porto (PT), in a local big chain supermarket (Pingo Doce S.A, Lisboa, Portugal), and they were preserved in the freezer at −18 °C until the experiments were carried out. Fruit packets were thawed overnight at fridge temperature (≈5 °C) for 8 h before their usage in the laboratory. One package of 250 g of frozen blueberries was used for each experiment. Once defrosted, fruits were washed under clean running water, and the extra juice resulting from thawing, which may have already been oxidized, was discarded.

The fruit juice was obtained by squeezing the whole blueberries using a high-performance, continuous juicer (Centrifugal Juicer Excel JE850, Kenwood, Hampshire, UK). Despite the juice yield obtained, an additional step of centrifugation was not used since excessive pulp was not retained, and the resulting juice was not too viscous and had a puree look. The percentage of juice yield was obtained as the difference between the final mass of blueberry juice divided by the initial mass of defrosted blueberries, as follows in Equation (1):

(1) $$Yield\;\left(\%\right)=\frac{mass\;of\;juice}{mass\;of\;defrosted\;blueberries}\times 100$$

All the blueberry packages showed an average yield of 61% after juicing.

The juices were immediately analyzed for fresh samples or thermosonicated or heat-treated. After analysis, the juices were discarded. All analyses were carried out in triplicate.

### 2.2. Treatments

#### 2.2.1. Thermosonication

A 50 mL sterilized beaker filled with 20 mL of freshly squeezed blueberry juice was sealed with parafilm and subjected to thermosonication treatment. In an open vessel plant design, a high-performance ultrasonic processor (Model CL-334, QSonica, Newtown, CT, USA) with a maximum power output of 700 watts applied ultrasonic waves at a constant frequency of 20 kHz. An ultrasonic probe of 13 mm in diameter was immersed in 1 cm of the juice. The generator has a touchscreen display through which it is possible to select the desired amplitude (0–100%), pulsation, and duration of the treatment. During the treatment, power (W) and energy (J) were continuously displayed on the screen. In turn, the beaker containing the juice was immersed in a thermostatic water bath (Thermomix BU circulator, B. Braun, Melsungen, Germany), with the juice being agitated once the desired temperature of the outside water was reached. The treatments were carried out at two different amplitudes, 60% and 100%, each at two different mild temperatures, 45 °C and 55 °C. The sonication was set up for a pulse period of 10 s on and 5 s off to allow effective sonication while preventing excessive heating. The treatments at 45 °C were submitted for 30 min, while the ones at 55 °C were carried out for 3 min. An additional temperature probe, separate from the ultrasonic one, was inserted into the juice sample and connected to the ultrasonic generator to continuously monitor the internal temperature of the blueberry juice and ensure it was maintained within ±1.0 °C throughout the entire procedure. The sonication treatment started once the experimental desired temperature was reached inside the juice sample. The internal temperature was continuously adjusted by adding ice to the water bath, as ultrasonication increases the samples’ temperature due to the cavitation phenomenon. All treatments (both for *L. innocua* inactivation and quality analyses) were replicated three times.

#### 2.2.2. Heat Treatments

Juice samples, referred to as controls, were heat-treated using a 50 mL glass beaker in a thermostatic water bath (Thermomix BU circulator, B. Braun). Treatments were carried out at the same temperatures of the thermosonicated juices, 45 °C and 55 °C. The heat treatment lasted 60 min for 45 °C and 10 min for 55 °C. The temperature was monitored with a digital timer log (Fantast, IKEA, Älmhult, Sweden) to verify that it was maintained during the experimental procedure ± 1.0 °C. All experiments (both for *L. innocua* inactivation and quality analyses) were carried out in triplicate.

### 2.3. Listeria Analyses

#### 2.3.1. *L. innocua* Culture, Inoculation, and Enumeration

The culture of *L. innocua,* strain 2030c was obtained from the Public Health Laboratory Service (PHLS) (Colindale, UK) private collection. To activate the culture, a loopful of thawed culture was streaked on Tryptic Soy Agar (TSA) (Biokar Diagnostic, Cedex, Pantin, France) with the addition of 0.6% Yeast Extract (TSAYE) and maintained in a laboratory fridge at 7 °C. A single colony from the strain was then drawn with a loop and transferred to a sterilized tube filled with 10 mL Tryptone Soy Broth (TSB) (Biokar Diagnostic, Cedex, Pantin, France) and incubated for 24 h at 37 °C to achieve the desired cell density (10^9^ CFU/mL).

**Figure 1:**
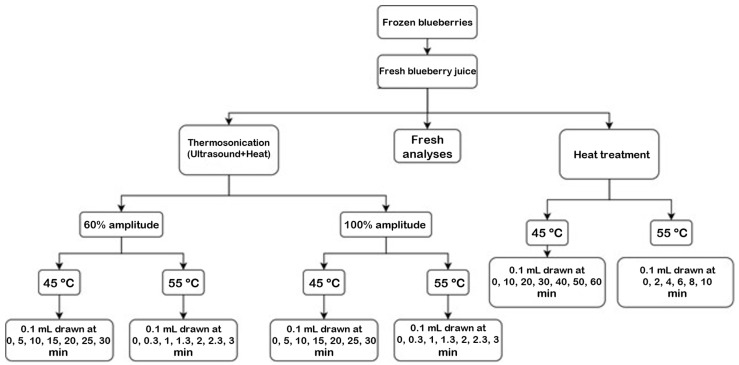
Time points for the various conditions analyzed refer to the microbiological part.

For each experiment, the second subculture of *L. innocua* was consistently prepared and incubated at 37 °C for 24 h to produce cultures in the stationary phase [13]. Juice samples were artificially inoculated by adding 1 mL of *L. innocua* suspension to 19 mL of blueberry juice, obtaining a final contamination of approximately 10^7^ CFU/mL. *L. innocua* was quantified in duplicate during both the thermosonication and heat treatments. This was conducted before and after each treatment using decimal dilutions, and the samples were spread on PALCAM agar with a selective supplement (BIOKAR Diagnostics, Pantin, France). All these microbiological procedures were conducted near multiple open flames to create a sterile environment and minimize airborne contamination. The plates were then incubated for 48 h at 37 °C. The viable *L. innocua* colonies were counted using the Plate Count Method, and the results are presented as CFU/mL of juice. The time points at which the juice volume were drawn are according to the process flow diagram shown in Figure 1.

#### 2.3.2. Modelling of *L. innocua* Inactivation Kinetics

The Weibull model, a statistical model for distributing microbial inactivation times, was used to fit all the logarithmic inactivations of *L. innocua*. It has been recognized that this model is very versatile and performs well in different processes compared to others. Therefore, its usage can improve food safety and quality [14]. When microbial inactivation follows a linear or non-linear behavior, the data experimentally obtained may be mathematically described by the Weibull model as in Equation (2):

(2) $$log\left(\frac{N}{N_{0}}\right)=-k\times t^{n}$$

where N is the blueberry juice microbial load (CFU/mL) at a specific time t (min) during the treatment, N_0_ is the initial N value (CFU/mL), k is the inactivation rate ($\min^{-n}$), and n is the shape parameter (dimensionless). A value of n = 1 demonstrates linearity, n < 1 indicates an upward concave curve, and n > 1 corresponds to a downward concave survival curve.

A least squares non-linear regression analysis was conducted with IBM SPSS Statistics 27 for MacOS (IBM, 2021, Armonk, NY, USA), and the parameters k and n were obtained.

The quality of the regressions was assessed by evaluating the randomness and normality of the residuals and the coefficient of determination R^2^. The estimated model parameters’ precision was determined by calculating confidence intervals at 95%.

### 2.4. Quality Analyses: Physicochemical Properties

#### 2.4.1. pH, Total Soluble Solids Content, and Water Activity

Before and after each treatment, the total soluble solids (TSSs, °Brix) content was analyzed using a digital refractometer (Digital Hand-held PAL-LOOP, Atago Co., Ltd., Tokyo, Japan). pH values were measured using a pH meter (BASIC 20, Crison Instruments, S.A., Barcelona, Spain).

Values for water activity (a_w_) of the blueberry juice were measured before and after treatment of the samples. A dew-point hygrometer (AquaLab 3TE, Decagon Devices Inc., Washington, DC, USA) was used at 22 ± 1 °C. Distilled water was used to calibrate the equipment until the value of $a_{w}$ was 1.000 ± 0.03. Three true replicates were carried out for all determinations.

#### 2.4.2. Color

Before and after treatments, the color of blueberry juice was measured in triplicate using a colorimeter (CR-400, Konica-Minolta, Osaka, Japan), where CIE Lab-scale (L*, a*, b*) values were measured. The analyses were carried out inside a photo studio box (HAVOX HPB-40D, 43 × 43 × 43 cm) artificially illuminated to avoid interference with external light. The box also contains a light-diffusing fabric to prevent reflections and ensure uniform lighting. Blueberry juice (around 5 mL; 7 mm thickness) was poured into a drilled plastic round container, and the colorimeter was placed on the container to take the values. The equipment was calibrated using a standard white reference tile. The L* value represents the color brightness coordinate, and it measures the color whiteness, with black as 0 and white as 100; a* is the coordinate for redness (when the value is positive, +60) and for greenness (when the value is negative, −60); b* is the coordinate that measures yellow when positive (+60) and blue when negative (−60). The Chroma value represents the color intensity, while the Hue angle measures and defines colors: 0° denotes red, 90° denotes yellow, 180° green, and 270° blue [12,15,16]. The total color difference (TCD) between the fresh samples and the thermosonicated and heat-treated ones was calculated according to Equation (3):

(3) $$\mathrm{TCD}=\sqrt{{\left(L_{0}^{*}-L^{*}\right)}^{2}+{\left(a_{0}^{*}-a^{*}\right)}^{2}+{\left(b_{0}^{*}-b^{*}\right)}^{2}}$$

where the 0 index stands for the values obtained from fresh juice.

Chroma and Hue angle were determined based on Equations (4) and (5):

(4) $$Chroma=\sqrt{a{*}^{2}+b{*}^{2}}$$

(5) $$\mathrm{Hue}\;\mathrm{angle}=\tan^{-1}\left(\frac{b*}{a*}\right)$$

### 2.5. Quality Analyses: Bioactive Compounds

#### 2.5.1. Total Phenolic Content

The blueberry juice samples’ total phenolic contents (TPCs) were evaluated using the Folin–Ciocalteau method [12,15,17]. To ensure the accurate measurement of phenolic compounds in blueberry juice, an extraction procedure was employed before the TPC analysis. This step aimed to enhance the release of phenolic compounds from the matrix, as preliminary tests indicated that direct sample dilution alone did not yield consistent results. The extraction protocol was therefore selected to increase assay sensitivity, as is commonly performed in studies of phenolic content in fruit juices. First, phenolic compounds were extracted by measuring 25 mL of blueberry juice and adding 50 mL of 100% methanol (Fisher Scientific, Leicestershire, UK). Afterward, the solution was homogenized with an ultra-turrax homogenizer (Ika digital T25, IKA^®^-Werke GmbH & Co. KG, Staufen, Germany) and centrifuged at 4 °C and 5000 rpm for 10 min. The procedure followed the method described by Fundo et al. [12], in which standard solutions with different concentrations of gallic acid were prepared. The main reaction was attained by combining the standard solution or juice sample with ${\mathrm{Na}}_{2}{\mathrm{CO}}_{3}$ 75 g/L, distilled water, and Folin–Ciocalteau reagent in a spectrophotometric cuvette. After that, the cuvette was agitated before being maintained in the dark for 1 h at room temperature. Absorbance was then read using a UV/VIS spectrometer (UNICAM PU-8625, Unicam Sistemas Analíticos Lda, Algés, Portugal) at 750 nm. The spectrometer was previously calibrated with 100% methanol. The absorbance values were measured in triplicate for each of the three extractions made for each juice sample. The final amount of total phenolics was reported as gallic acid equivalent (GAE) per liter of sample (mg/L) using a gallic acid standard curve prepared using different gallic acid concentrations.

#### 2.5.2. Total Anthocyanin Content

The total monomeric anthocyanin content (TAC) of blueberry juice samples was analyzed using the pH differential method suggested by AOAC [5,15,18,19,20,21]. The method is based on the structural change of monomeric anthocyanin chromophores between pH 1.0 and 4.5. These anthocyanins undergo a reversible structural transformation as a function of pH, becoming colored at pH 1.0 and colorless at pH 4.5. Therefore, the pigment absorbance difference at $\lambda_{vis-max}$ (around 520 nm) is directly related to the pigment concentration in the sample.

First, two buffer solutions were prepared: pH 1.0 ± 0.5 buffer (potassium chloride, 0.025 M), made with 1.86 g KCl (Cloreto Potassio, Pronalab, Lisbon, Portugal), 980 mL of distilled water, and around 6.5 mL of HCl (Hydrochloric acid fuming 37%, Merck KGaA, Darmstadt, Germany); pH 4.5 ± 0.5 buffer (sodium acetate, 0.4 M), prepared with 54.43 g of ${\mathrm{CH}}_{3}{\mathrm{CO}}_{2}\mathrm{Na}\cdot 3H_{2}O$ (Sodium acetate trihydrate, Sigma-Aldrich, Merck KGaA, Darmstadt, Germany)), 960 mL of distilled water, and around 15 mL of HCl. Afterward, in spectrophotometric cuvettes, 1000 µL of juice samples was diluted with 2.4 mL of the two buffer solutions pH 1.0 and 4.5 according to a dilution factor of 25. Cuvettes were then agitated and incubated in the dark at room temperature for 20 min. The absorbance of these dilutions (both at pH 1.0 and 4.5) was read at 520 nm and 700 nm using a UV/VIS spectrometer (UNICAM PU-8625, Unicam Sistemas Analíticos Lda, Algés, Portugal). Three true replicates were carried out for all determinations. The absorbance value was calculated as follows in Equation (6):

(6) $$A={\left(A_{520}-A_{700}\right)}_{pH1.0}-{\left(A_{520}-A_{700}\right)}_{pH4.5}$$

in which $A_{520}\;$is the value measured at 520 nm in the samples diluted with pH 1.0 or pH 4.5 buffers; $A_{700}$ is the absorbance value assessed at 700 nm in the samples diluted with pH 1.0 or pH 4.5 buffers.

The TAC was calculated as in Equation (7) and expressed in mg/L:

(7) $$TAC=\frac{\left(A\times MW\times DF\times 1000\right)}{\varepsilon \times l}$$

where A is the absorbance value previously calculated, MW is the molecular weight of cyanidin-3-glucoside (449.2 g/mol), DF is the dilution factor (25), ε is the molar extinction coefficient (26,900 L${\;\mathrm{mol}}^{-1}{\mathrm{cm}}^{-1}$), and l is the cuvette path length (1 cm).

### 2.6. Quality Analyses: Rheological Evaluation

The rheological behavior of blueberry juice samples was determined through analyses conducted on a Gemini Advanced Rheometer (Bohlin Instruments, Malvern, Worcestershire, UK) coupled with a Peltier unit for temperature control (at 25 °C) and using a 4°/40 mm stainless steel cone-and-plate geometry probe. First, 800 µL of juice was poured with a pipette on the sampling plate, and the analysis was performed using a 150 µm gap. Shear stress (τ) was determined as a function of shear rate (γ), which varied between 0.001 and 10 $s^{-1}$. The power law model (Equation (8)) parameters, *n* and *k_visc_*, were obtained by least squares non-linear regression of the shear stress versus shear rate:

(8) $$\tau =k_{visc}\times \gamma^{n}$$

In the above, the flow behavior index is *n*, and *k_visc_* is the coefficient for consistency (Pa $s^{n}$).

Analyses were conducted in three independent samples (three measurements were performed for each sample).

### 2.7. Statistical Analysis

Differences between several attributes of blueberry juices were statistically analyzed using a one-way ANOVA, including all the data obtained from independent and repeated experiments. The normality of data and homogeneity of variances were measured using Shapiro–Wilk and Levene’s tests, respectively. The Tukey post hoc test was applied to assess which specific groups’ means are significantly different from each other. The significance level assumed in all tests was 5%. IBM SPSS Statistics 27 for MacOS (IBM, 2021, Armonk, NY, USA) was used in all data analyses.

The data results are expressed as mean ± confidence intervals at 95%.

## 3. Results and Discussion

### 3.1. L. innocua Inactivation

*L. innocua* is a closely related species of the pathogenic *L. monocytogenes.* They are found in the same food products, mainly ready-to-eat and minimally processed foods. Listeriosis, meningoencephalitis, spontaneous abortion, stillbirth, and septicemia are some of the implications of the growth of *L. monocytogenes*. At the same time, *L. innocua* does not represent a hazard to human health, and it has been used several times in thermal inactivation studies [22]. *L. monocytogenes* is not a relevant fresh fruit juice pathogen. However, this pathogen and its surrogate show psychotropic behavior, growing on equipment surfaces and in adverse conditions [23,24]. Therefore, they are of concern in fresh fruit and fruit juices. *Listeria* has been shown to grow and survive in oranges during refrigeration and abusive storage conditions. This is why a study on *Listeria* in blueberry juice, which also has a low pH, was needed.

**Figure 2:**
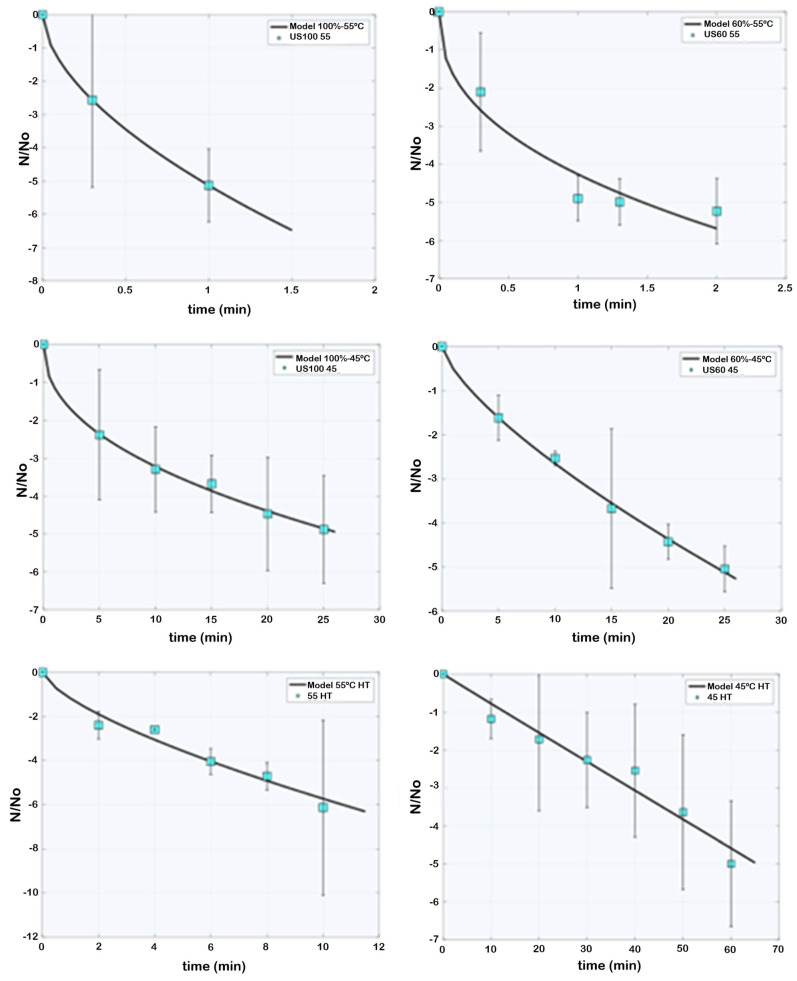
Log-survival data of *L. innocua* in blueberry juice submitted to thermosonication and heat treatments. Points are the mean values, and the bars are confidence intervals at 95%. Lines represent the Weibull model fits (Equation (2)).

The results of *L. innocua* inactivation in blueberry juice are reported in Figure 2. Log reductions (log*N*/$N_{0}$) of *L. innocua* are shown for each treatment. The Weibull model (Equation (2)) was adequate in each case in fitting the experimental inactivation data of *L. innocua*, as shown in Figure 2.

The resulting graph indicates that in the control sample treated at 55 °C (55 HT), a reduction of 4.7 log cycles of *L. innocua* was obtained after 8 min and a decrease of 6.1 logs after 10 min. In the control sample treated at 45 °C (45 HT), a 4.9 log cycle reduction of *L. innocua* was observed after 60 min. At 55 °C, the decrease in the survival of the bacteria was higher than at 45 °C. The inactivation line of the sample treated at 45 °C shows an almost linear inactivation.

The first conclusion that can be revealed is that higher log reductions are reached in a shorter time when sonication is added to the heat treatment. With a 100% amplitude sonication added to the 55 °C treatment (US100 55), a 5.1 log reduction was achieved right after 1 min. The same happened with the sample treated with a 60% amplitude sonication added to the 55 °C treatment (US60 55), where 4.9 and 5.2 log reductions were achieved after 1.3 and 2 min, respectively. With 100% and 60% amplitude sonications added to the 45 °C treatments (US100 45 and US60 45), 4.9 and 5.0 log reductions were achieved after 25 min, respectively. This difference in processing times to achieve a 5-log reduction highlights the effectiveness of sonication coupled with heat compared to the heat treatment alone. From these results, it is also easily understandable that the difference in sonication amplitude does not play a role in the inactivation behavior of *L. innocua* since the process times needed to inactivate the bacterium are equal in both US100 55 and US60 55 and US100 45 and US60 45.

The literature has also widely recommended the use of thermosonication compared to ultrasonication alone. As already reported, to increase the inactivation rate of *E. coli* in black mulberry juice, the preferred treatment was thermosonication, as a temperature increase is generally accompanied by a further decrease in the number of microorganisms [18]. Relatively high temperatures contribute to further weakening of the microbial cell membrane and cause extensive cell damage that is already ruined by cavitation. Furthermore, there is a reduction in the processing temperature and time compared to ultrasonication or heat treatment alone, making the process more economically feasible [25,26].

From the Weibull model, kinetic parameters were estimated for all the studied conditions (Table 1). As can be noted in Table 1, all the coefficient of determination (R^2^) values were high for all cases, varying from 0.87 to 0.97.

The parameter k represents the *L. innocua* inactivation rate or the reciprocal of the first decimal reduction time. As observed, k depends on the temperature, process, and treatment amplitude. For the same treatment, as temperature increases, k also increases. For the same temperature and comparing the thermosonication treatments at different amplitudes, k increases with the rise in thermosonication amplitude. Another conclusion is that the inactivation is faster in thermosonicated treatments than in heat treatments since lower k values were attained for these last ones.

The parameter n represents the shape of the survival curves. As shown in Table 1, all the conditions studied presented an n parameter lower than 1, which indicates an upward concavity of all the log-survival curves; there is an exception for 45 HT, which showed close to linear behavior (0.99 ± 0.30). The ascending concave profile suggests the presence of a tail in each of the survival curves. The observed tailing phenomenon is due to the presence of a thermotolerant subpopulation of cells (Figure 2).

The results were satisfactory since, as stated before, the Food and Drug Administration describes a juice as stable in which at least 5-log reductions of the target microorganisms have been attained after applying a preserving technology [27]. In all of the thermosonicated samples, a 5-log reduction was achieved relatively quickly, especially in the samples sonicated at 55 °C.

The preferred treatment for *L. innocua* inactivation in blueberry juice was US100 55, as it showed the highest k-value, meaning less time is needed to attain a 1-log reduction. Furthermore, the treatment presented the shortest processing time to inactivate 5-log *Listeria* cells (1 min).

The presented results are closed to the ones reported in raw milk inoculated with *L. innocua* and treated with different amplitudes (60, 90, and 100%) at a constant temperature of 63 °C for 30 min, obtaining a final decrease in the cell loads higher than 5-log reductions. As in the current study, it was observed that different amplitudes (intensities 60, 90, and 100%) had similar behavior throughout inactivation. The effectiveness of thermosonication against conventional thermal treatments also emerged [28].

Other studies also showed an enhancement in bacteria inactivation when coupling ultrasonication and thermal treatment. Tremarin et al.’s work showed a higher inactivation of *A. acidoterrestris* spores in apple juice when the heat treatment was paired with sonication [7]. In other research, heat treatment alone was insufficient to decrease the 5-log cycles of *E. coli* O157:H7 in blueberry juice. However, thermosonication increased the bacterium inactivation (5.10-log reduction at 60 °C) [5].

### 3.2. Characterization of Fresh Blueberry Juice

Untreated blueberry juice was analyzed right after the squeezing with the juicer for pH, TSSs, $a_{w}$, color, TPC, and TAC. The values are summarized in Table 2.

The most protective effects against chronic diseases shown by berry cultivars are due to their bioactive molecules, including anthocyanins and other phenolic compounds [29,30]. It has been demonstrated that blueberry extracts could heavily influence the inhibition of the proliferation of cancer cells: for 24 to 68% of breast cancer cells and for 46% to 74% of colon cancer [31]. Generally, it has been observed that among quality indicators, the juice color and the bioactive compounds are the most affected by berry juice processing [21].

The pH value for the untreated blueberry juice was 3.08 ± 0.28. This result was similar to the ones described by Medina-Meza et al. [20] (3.81 ± 0.00), Režek Jambrak et al. [32] (3.50 ± 0.00), Mohideen et al. [15] (3.12 ± 0.02), and Zou and Hou [33] (3.12 ± 0.02). Differences in raw pH depend on which blueberry cultivar has been used for the experiments [34].

The total soluble solids content (TSSs) in the fresh juice was 12.43 ± 1.85. Zou and Hou [33] presented 5.0 ± 0.01 as a result of their fresh sample, and this marked difference can be brought back to the extra filtration step they used in their sample preparation. For basic fruit juices, meaning those with no addition of another kind of fruit juice that are not concentrated, the TSSs (°Brix) should be the same as the original fruit. This cannot be changed unless other juice from the same fruit is added to the blend. If a juice instead needs to be reconstituted, the Brix level must be in accordance with the minimum level established by the Codex Alimentarius in the Annex. For blueberry (*Vaccinium myrtillus* L.), the Codex Alimentarius states that it needs to be 10.0 [35].

The fresh blueberry juice’s total phenolic content (TPC) was 495.48 ± 7.07 mg GAE/L. This value was similar to the one presented by Zou and Hou, 528 ± 9 mg/L [33].

The untreated blueberry juice’s total anthocyanin content (TAC) was 272.37 ± 34.3 mg/L. This value was lower than the results obtained by Mohideen et al. [15] (408.29 ± 21.6 mg/L) and Medina-Meza et al. [20] (around 650 mg/L), while it was higher than the ones analyzed by Zou and Hou [33] (228 ± 8 mg/L).

Recently, it has been shown that pulsed ultrasound treatment can better preserve texture, color, ascorbic acid, and total soluble solids compared to continuous ultrasound [36].

The processing times needed to reach the 5-log reductions of *L. innocua* in each treatment were used to further thermosonicate or heat-treat the samples to assess their quality parameters. Therefore, the samples were sonicated with 100% and 60% amplitude at 55 °C for 1 min and with 100% and 60% amplitude at 45 °C for 25 min. The thermally treated samples followed a processing time of 10 min (55 °C) and 60 min (45 °C).

### 3.3. Physicochemical Properties

#### 3.3.1. pH, TSSs, and a_w_

Berries and fruit juices are part of the high-acid fruits group, which contain different organic acids, such as citric acid, malic acid, tartaric acid, and fumaric acid. Therefore, pH is an essential antimicrobial in blueberry juice, as acidity is the most critical factor affecting microbial spoilage. However, after treatments, pH can vary, and it is no longer a barrier for pathogens, so it is essential to check juices’ pH values.

**Figure 3:**
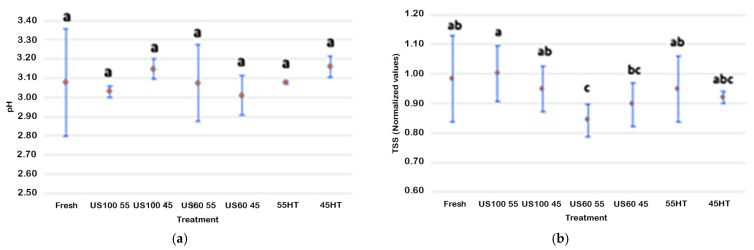
Effects of ultrasound and heat treatments on the pH (**a**) and TSSs (**b**) of blueberry juice. Points are the mean values, and the bars are confidence intervals at 95%. Different lower-case letters show a significant difference (*p* < 0.05).

As shown in Figure 3a, no significant difference in pH was noted for any treatment, regardless of ultrasonic intensity or temperature (*p* < 0.05). However, it is important to mention that the untreated sample showed some variability in this parameter, as can be observed by the higher confidence interval. Nevertheless, this result is in accordance with other studies on other juices, indicating that US treatment did not cause a further liberation of organic acids in the juice [10,33,37,38].

As among the fresh samples, the °Brix values differed based on each batch. Therefore, values were normalized to decrease some of this variability, probably due to different internal fruit maturation. As shown in Figure 3b, the US and heat-treated results were similar regarding the TSSs of the fresh juice. However, the TSSs of blueberry juice US60 55 were significantly lower than the untreated juice and 55 HT, US100 55, and US100 45. In other words, an enhancement in TSSs was seen in the thermosonicated samples, maybe because sonication destroys cell walls and fruit tissues. As this happens, more water can enter fruit cells and, therefore, more soluble solids cross the cell membranes [33].

It was observed that in both the thermosonicated and control samples, there were no prominent (*p* < 0.05) variations in water activity values. Therefore, all blueberry juice samples presented an $a_{w}$ average of 0.99 ± 0.001. Water activity measures the free or available water in products supporting microbial growth and spoilage processes [12].

#### 3.3.2. Color

In a product, color is an essential factor in consumers’ choices, and sometimes it even plays a more critical role than the flavor itself. Understanding how the color changes over different treatments is of great interest [39]. In many studies, cavitation resulting from the ultrasound process is the cause of color changes in juices and nectars [18,32,40,41]. The impact of thermosonication and heat on blueberry juice color is reported in Table 3.

This study’s initial chromatic value L* was 16.10 ± 2.16. By observing Table 3, it can be concluded that this parameter significantly changed after US100 55, US60 55, and 55 HT. In accordance with Zou and Hou’s [33] work, L* increased after sonication, enhancing the lightness of the blueberry juice. However, it has also been shown that continuous sonication alone, regardless of the amplitude, does not influence the L* values of blueberry juice [15]. Therefore, this means that the L* parameter depends on temperature, increasing with its increment. The results are similar to those of others who noticed a significant increase in L* and a* values after thermosonication, especially at higher processing times and amplitude levels [42]. The observed increase in L* values can be caused by the homogenization effect of sonication [43].

The chromatic value a* of the fresh sample is 4.07 ± 1.67, which denotes redness. It increased only with thermosonication at 55 °C (US100 55 and US60 55). This chromatic result is given by anthocyanin’s presence, which enhances the juice’s aesthetic perception, contributing to its attractiveness and desirability regarding consumers. According to another study, a decrease in the a* value is related to anthocyanin degradation and the formation of Maillard reaction products in berry juices [44].

The color b* coordinate of the fresh juice was 1.55 ± 0.35, which indicates yellowness. It was also significantly increased with thermosonication at 55 °C (US100 55 and US60 55) and due to both heat treatments (45 and 55 °C).

The Chroma value was initially 4.36 ± 1.68, while after thermosonication and heat treatments at 55 °C, its values significantly increased (*p* < 0.05), showing an increased saturation and, therefore, a more vivid color [45], which was also very noticeable to the naked eye.

A slight increase in the Hue angle, initially 0.37° ± 0.07°, denoting red, can be noticed in Table 3, especially in the heat-treated samples. This can be attributed to the degradation of phenolic compounds [46].

Other authors also observed significant increases in all color values in their works on sonicated and thermosonicated mulberry juice samples [15]. Some researchers also suggested that the presence of the pulp in their sonicated orange juice increased the difference in color after thermosonication compared to their samples without pulp [9].

**Figure 4:**
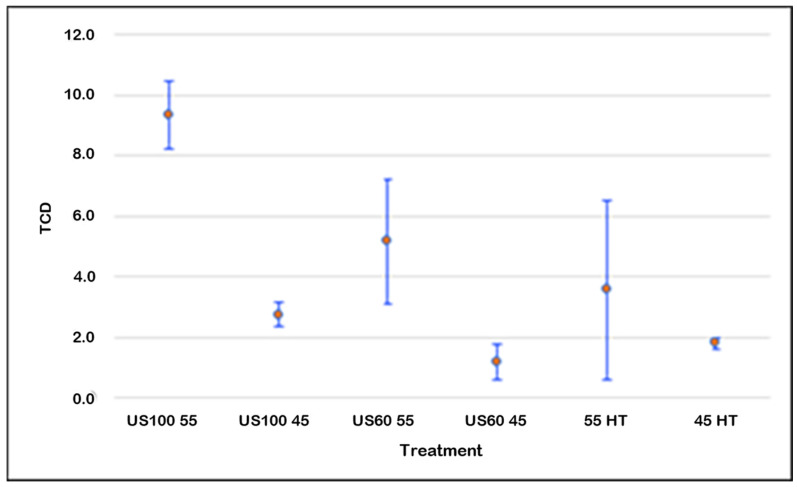
Effects of ultrasound and heat treatments on blueberry juice (TCD) color. Points are the mean values, and the bars are confidence intervals at 95%.

The total color differences (TCDs) can be seen in Figure 4. The classification of the differences in visual color is based on the TCD. If the values of TCD are >2, this corresponds to a prominent difference in the visual perception of the juice [47]. According to DrLange’s [48] scale, a value of the total color difference between 1.5 and 3 is considered “distinct” from the fresh sample. As shown in the graph, the treatment that mostly retained the original color of the fresh juice was US60 45, followed by the control 45 HT. The TCD of treatment US100 45 was slightly higher than 2. However, the treatments that greatly affected the color of blueberry juice were US100 55 and US60 55, followed by the control sample 55 HT. This underlines that the increasing amplitudes did not majorly impact the color (even if there are differences between the TCD of US100 55 and US60 55 and between US100 45 and US60 45), but that color was mainly impacted by increasing the temperatures. Samples sonicated at 100% and 60% at 45 °C showed less change in color. Significant color changes are pointed out as unfavorable, as they are part of a negative sensory impact of the treatment. These results are similar to the ones showed by Rawson et al. [42] on thermosonicated watermelon juice.

### 3.4. Bioactive Compounds

#### 3.4.1. Total Phenolic Content

Phenolic compounds are secondary metabolites found in high quantities in fruit and are mainly located on the epidermal layers of fruit peels and seeds. They are known to be essential bioactive compounds that play a major role in the biological activities of products derived from many fruits. Therefore, promising chemical compounds and their bioactivity in commercial juices need to be evaluated, as it is essential to provide consumers with knowledge about the health benefits of the products [49]. Blueberries have the highest percentage (about 70%) of soluble phenolics [50]. Mostly, all the benefits are related to the antioxidant compounds found in blueberry juice, such as flavonoids (anthocyanins and proanthocyanins), resveratrol, and phenolic acids, among others [51].

**Figure 5:**
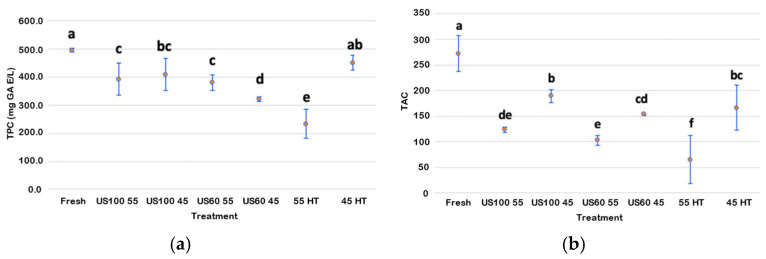
Effects of ultrasound and heat treatments on total phenolic contents (**a**) and anthocyanins (**b**). Points are the means values, and the bars are confidence intervals at 95%. Different lower-case letters show a significant difference (*p* < 0.05).

The total phenolic content (TPC) results are shown in Figure 5a for the fresh, thermosonicated, and heat-treated juices. As noted, the only juice that presented a not significantly different phenolic content compared to the untreated juice was the one heat-treated at 45 °C. Even though the temperature was low, it allowed significant retention of these bioactive compounds (91%). The lowest retention of phenolics (47%) was in the heat-treated juice at 55 °C. Therefore, this suggests that temperature is the leading cause of degradation of phenolic compounds. However, from Figure 5a, an interesting difference emerges between the TPC of 55 HT and that of US100 55 and US60 55: even if the temperature used during the thermosonication was the same as in the heat treatment, a higher retention of the phenolic compounds was achieved with thermosonication (79% in US100 55 and 76% in US60 55). Regarding the thermosonication at 45 °C, the results surprisingly showed slightly a lower retention of polyphenols in the sample treated at 60% amplitude (65%). The sample treated at 100% amplitude was the thermosonicated sample with the most phenolic retention (82%). These results could also be related to the short time used in the thermosonication process at 55 °C (1 min) compared to the 25 min used for the thermosonication at 45 °C. According to Rawson et al. [42], a significant degradation in the TPC at higher thermosonication times in watermelon juice was seen. Only the variation in amplitude at 55 °C did not show differences in retaining these bioactive compounds.

These results are similar to the work of Saeeduddin et al. [52] on pear juice. When the juice was thermosonicated at 25 °C, an increase in phenols was detected, while at higher temperatures (45 and 65 °C) these compounds were lost. However, the loss was less than conventionally pasteurized samples. Nonetheless, in the current study, the 45 °C thermosonicated samples did not enhance the TPC compared to the higher-temperature ultrasonicated blueberry samples.

Other studies reported different conclusions. According to the work of Medina-Meza et al. [20], the TPC was not significantly different between fresh blueberry juice and sonicated samples. On the other hand, Zou and Hou [33] observed that polyphenols in blueberry juice were enhanced with sonication alone, especially with the increasing process time. In another study, the TPC in blueberry juice increased with flow rate (continuous sonication) and amplitude [15].

This behavior in polyphenols was also observed in other studies, showing that sonication alone could improve bioactive compounds in juices [33,53,54]. The reason has been described as a cause of cavitation created by the ultrasonic process: it causes mechanical disruption of the cell walls of the suspended particles in the juice, releasing the bound form of phenolic acids present inside [52,55].

#### 3.4.2. Total Anthocyanin Content

Anthocyanins (TAC) are water-soluble natural pigments that belong to the class of health-promoting phenolic compounds with flavonoids and tannins. Studies have revealed that anthocyanins can protect the human organism against oxidative stress, showing free radical scavenging properties [56,57]. As the color of fruit depends on anthocyanins, it is a primary fruit sensory characteristic that directly correlates with nutritional quality. However, it is well known that anthocyanins are unstable compounds that are easily degraded by treatments. The anthocyanin content in blueberry fruit is very high, accounting for more than half of the TPC [58].

As shown in Figure 5b, all the treatments significantly decreased the TAC compared to the fresh juice. However, the most adverse effect occurred on the blueberry juice control sample treated at 55 °C, followed by US60 55 and US100 55, indicating the strong impact of temperature on bioactive compounds. The thermosonicated sample that retained anthocyanins better (69%) was US100 45, followed by sample US60 45, with a slight difference in retention (56%). In the anthocyanin measurements, again, a minor difference in retention due to the different amplitudes seemed to exist at 45 °C, as also shown by the variations in values of Tiwari [59].

Another study showed, instead, a very high retention (>94%) of anthocyanins in pulsed-sonicated blackberry juice at 100% amplitude and a constant frequency of 20 kHz [55]. Once more, this can be explained by the fact that only sonication was used, and heat was not applied. In another article, it was suggested that the temperature be kept below 40 °C during thermosonication to enhance the extraction of anthocyanins in berries [60].

Many works that used sonication alone agree with our results, showing a reduction in anthocyanins in sonicated samples [20,61]. As reported in other studies, the degradation of anthocyanins is due to the cavitation effect. This process causes an increase in heat and pressure, which leads to the reduction of the bioactive compounds, as is also the case in pulsed sonication [18,59].

### 3.5. Rheological Properties

The rheological parameters of the juices, obtained from the Power Law model (k_visc_ and n) [20], are included in Table 4. The results revealed a non-Newtonian behavior for all blueberry juices, as n was lower than 1 in all cases, indicating a shear-thinning behavior. This places them in the range of pseudoplastic fluids. Therefore, the juices present a low apparent viscosity at higher shear rates across the shear rates 0.001 and 10 $s^{-1}$. The flow behavior index did not show significant differences (*p* > 0.05) after the thermosonication and heat treatments, revealing that the behavior of the juice did not change with sonication or heat.

These results are in accordance with Medina-Meza’s research, which showed a shear-thinning behavior in a blueberry puree. However, the rheology of the blueberry puree was dramatically modified by ultrasonication treatment, showing a progressive shift from a non-Newtonian to a Newtonian model [20].

Our results are in contrast with the n values observed by Šimunek et al. [62], who found that blueberry juice showed a non-Newtonian dilatant fluid characteristic (n > 1) in all samples of untreated, pasteurized, and sonicated blueberry, apple, and cranberry juices, while the consistency coefficient k increased and decreased based on the temperature applied.

The consistency coefficient k_visc_ showed no differences with the fresh juice in the US10055 and US60 45 samples, while it showed a slight but significant decrease (*p* < 0.05) after treatments US100 45 and US60 55. Interestingly, the highest increase in the consistency coefficient was seen after the 45 HT treatment, and this may be explained by the longest process time that the sample underwent (60 min).

In Šimunek et al.’s [62] work, the consistency coefficient had the most significant increase in apple juice after ultrasonic treatment at 60% amplitude and 20 °C for 3 min (3.846 $\times 10^{-5}{\mathrm{Pa}\;s}^{n}$), while the most considerable decrease was detected after the treatment at 60% amplitude and 60 °C for 3 min (1.03 $\times 10^{-5}\;\mathrm{Pa}\;s^{n}$), showing how the impact of the temperature on viscosity is different according to each temperature range. For the blueberry juice, the largest increase in k (2.919 $\times 10^{-5}\;\mathrm{Pa}\;s^{n}$) was detected after sonication at 120% amplitude and 40 °C for 6 min, while a major decrease in k (1.109 $\times 10^{-5\;}\mathrm{Pa}\;s^{n}$) was seen in the sample treated at 60% amplitude and 60 °C for 3 min. Therefore, there is no specified correlation between the high temperature of the treatment and the viscosity of blueberry juice. However, in the pasteurized juices, the consistency coefficient decreased significantly compared to the untreated juices.

It has been shown that at low concentrations (below 30 °Brix), the viscosity of samples hardly changes [33,62,63]. Previous studies demonstrated that in colloidal solutions, the sugar concentration is connected with the viscosity, and the use of sonication causes the release of sugar compounds, increasing viscosity [41,64]. Therefore, if the concentration of the soluble sugars is low, the changes in viscosity will be limited. It was shown that the specific viscosity increases in a colloidal dispersion of solids with the rise in the particle–sugar interactions [65]. Therefore, the differences in rheological properties can be attributed to the differences in the sugar composition of different fruits, like in Simunek’s work on apples and blueberries, as apples contain higher soluble solids and sucrose values [62].

## 4. Conclusions

This study underscores the potential of thermosonication as an effective method for enhancing blueberry juice’s microbial safety and quality retention. By combining ultrasonication with mild heat treatment, we achieved significant inactivation of *L. innocua* while preserving the juice’s desirable qualities. The presence of phenolic compounds and anthocyanins, essential antioxidants, was notably retained, aligning with consumer trends toward healthier beverage options. The Weibull model effectively described the inactivation kinetics, underscoring the efficiency and rapid action of sonication treatments, with results showing that temperature played a more critical role than amplitude in influencing microbial inactivation and quality preservation.

The findings reveal that compared to conventional heat treatments, ultrasonication at both 60% and 100% amplitudes combined with temperatures of 45 °C and 55 °C enhanced microbial inactivation without compromising quality. This aligns with the growing consumer demand for minimally processed foods that retain their nutritional and sensory properties, suggesting the broad applicability of thermosonication to other fruit juices and liquid foods.

Future research should investigate the shelf life of thermosonicated blueberry juice, examine additional quality parameters such as antioxidant activity and ascorbic acid content, and assess enzyme activity (PPO and POD). Expanding the analysis using HPLC to profile specific anthocyanins will further clarify thermosonication’s impact, supporting its development as an effective, quality-preserving preservation method.

## Figures and Tables

**Table 1:** Weibull model parameters (k and n) and coefficient of determination $R^{2}$ for the different treatments applied on blueberry juice.

Treatment	k (min^−n^)	n	$R^{2}$
US100 55	5.13 ± 0.83	0.57 ± 0.30	0.94
US 100 45	1.15 ± 0.49	0.45 ± 0.15	0.92
US60 55	4.26 ± 0.36	0.42 ± 0.15	0.94
US 60 45	0.51 ± 0.17	0.72 ± 0.11	0.97
55 HT	1.18 ± 0.33	0.69 ± 0.14	0.96
45 HT	0.08 ± 0.09	0.99 ± 0.30	0.87

**Table 2:** Quality parameters of fresh samples.

Quality Parameter	Values
pH	3.08 ± 0.28
TSSs (°Brix)	12.43 ± 1.85
$a_{w}$	0.99 ± 0.01
Color Parameters	
L*	16.10 ± 2.16
a*	4.07 ± 1.67
b*	1.55 ± 0.35
Chroma	4.36 ± 1.68
Hue angle (°)	0.37 ± 0.07
Total Phenolic Content (mg GAE/L)	495.48 ± 7.07
Total Anthocyanin Content (mg/L)	272.37 ± 34.29

Values are mean ± confidence level at 95% of the three replicates.

**Table 3:** Color parameters of fresh, thermosonicated, and heat-treated samples.

Treatment	Color Parameters				
	L*	a*	b*	Chroma	Hue Angle (°)
Fresh	16.10 ± 2.16 ^ab^	4.07 ± 1.67 ^a^	1.55 ± 0.35 ^a^	4.36 ± 1.68 ^a^	0.37 ± 0.07 ^ab^
US100 55	21.38 ± 3.19 ^d^	11.25 ± 0.61 ^c^	4.17 ± 1.15 ^c^	12.00 ± 0.85 ^d^	0.35 ± 0.08 ^a^
US100 45	18.42 ± 0.59 ^bc^	5.15 ± 1.56 ^a^	2.36 ± 0.86 ^ab^	5.68 ± 1.03 ^ab^	0.43 ± 0.26 ^ab^
US60 55	20.03 ± 1.69 ^cd^	7.12 ± 1.64 ^b^	2.90 ± 1.07 ^b^	7.69 ± 1.82 ^c^	0.39 ± 0.09 ^ab^
US60 45	15.92 ± 2.31 ^a^	4.52 ± 1.02 ^a^	2.14 ± 1.17 ^ab^	5.02 ± 1.18 ^ab^	0.44 ± 0.20 ^ab^
55 HT	19.04 ± 2.63 ^c^	5.61 ± 1.76 ^ab^	2.78 ± 0.43 ^b^	6.27 ± 1.63 ^bc^	0.46 ± 0.12 ^ab^
45 HT	14.77 ± 0.38 ^a^	4.49 ± 1.04 ^a^	2.63 ± 0.29 ^b^	5.21 ± 1.00 ^ab^	0.53 ± 0.08 ^b^

Values are mean ± confidence level at 95% of the three replicates. Different lower-case letters show a significant difference (*p* < 0.05).

**Table 4:** Power Law model of rheological parameters of blueberry juice data for each process.

Treatment	$k_{\mathrm{visc}}\;(Pa\;s^{n}$)	n	$R^{2}$
Fresh	0.74 ± 0.42 ^ab^	0.21 ± 0.05 ^a^	0.95
US100 55	0.67 ± 0.11 ^ab^	0.29 ± 0.11 ^a^	0.95
US100 45	0.40 ± 0.43 ^b^	0.26 ± 0.70 ^a^	0.99
US60 55	0.38 ± 0.92 ^b^	0.27 ± 0.15 ^a^	0.98
US60 45	0.72 ± 0.41 ^ab^	0.19 ± 0.04 ^a^	0.97
55 HT	0.71 ± 0.17 ^ab^	0.32 ± 0.02 ^a^	0.95
45 HT	1.41 ± 1.80 ^a^	0.24 ± 0.01 ^a^	0.94

Different lower-case letters show a significant difference (*p* < 0.05).

## Data Availability

The original contributions presented in the study are included in the article, and further inquiries can be directed at the corresponding author.
